# Finding formulas: Does active search facilitate appropriate generalization?

**DOI:** 10.1186/s41235-021-00316-y

**Published:** 2021-07-19

**Authors:** Nicole R. Hallinen, Lauren N. Sprague, Kristen P. Blair, Rebecca M. Adler, Nora S. Newcombe

**Affiliations:** 1 Microsoft, 1 Microsoft Way, Redmond, WA 98052 USA; 2 Department of Psychology, Florida State University, 1107 W. Call St., Tallahassee, FL 32304 USA; 3 Stanford Graduate School of Education, 485 Lasuen Mall, Stanford, CA 94305 USA; 4 Department of Psychology and Human Development, Vanderbilt University, 230 Appleton Place, Peabody #552, Nashville, TN 37203-5721 USA; 5 Department of Psychology, Temple University, 1701 N. 13th Street, Weiss Hall, Room 318, Philadelphia, PA 19122 USA

**Keywords:** Induction, Transfer, Mathematics learning, Sketching, Algebra

## Abstract

**Background:**

One criterion of adaptive learning is appropriate generalization to new instances based on the original learning context and avoiding overgeneralization. Appropriate generalization requires understanding what features of a solution are applicable in a new context and whether the new context requires modifications or a new approach. In a series of three experiments, we investigate whether searching for an algebraic formalism before receiving direct instruction facilitates appropriate generalization.

**Results:**

(1) Searching buffers against negative transfer: participants who first searched for an equation were less likely to overgeneralize compared to participants who completed a tell-and-practice activity. (2) Likelihood of creating a correct new adaptation varied by performance on the searching task. (3) Asking people to sketch alleviated some of the negative effects of tell-and-practice, but sketching did not augment the effect of searching. (4) When participants received more elaborate tell-and-practice instruction, the advantages of searching were less notable.

**Conclusions:**

Searching for an algebraic formula prior to direct instruction may be a productive way to help learners connect a formula to its referent and avoid overgeneralization. Tell-and-practice instruction that only described the mathematical procedures led to the greatest levels of overgeneralization errors and worst performance. Tell-and-practice instruction that highlighted connections between the mathematical structure of the formula and the visual referent performed at similar or marginally worse levels than the search-first conditions.

## Introduction

Mathematics provides a way to formalize our descriptions of patterns observed in the world and define their scope of application. A central goal of mathematics instruction is to help learners develop flexible formalizations that they can transfer appropriately across contexts (Day & Goldstone, 2012; Star & Newton, 2009). Ideally, students should be able to recognize when to apply ideas and recognize the boundaries of when ideas are no longer appropriate. Broadly speaking, there are two potential approaches to acquiring flexible understanding of mathematical relations.

### Knowledge from empirical experience

In an induction-first approach, people learn ideas from experiences. This idea has roots in Piaget’s characterization of children as scientists learning from the world around them (1973), and more recently in embodied cognition research that emphasizes the role of perceptual-motor experience in learning (Abrahamson, 2009; Barsalou, 1999; Glenberg, 1997; Lakoff & Núñez, 2000). Instructional methods that come out of this tradition emphasize grounding symbols in hands-on activities (e.g., Lehrer & Schauble, 2004; Montessori, 1965). An array of discovery learning approaches begin with experiential activities and transition to more formal notation through inductive reasoning (Fuson et al., 1997; Hiebert et al., 1996; Kamii & Dominick, 1998). In particular, inventing a symbolic representation based on instances provides students with a better sense of the relationships among variables (Rittle-Johnson & Star, 2007; Schwartz et al., 2011). These approaches lead to improvements in students’ conceptual knowledge and transfer (Carpenter et al., 1998; Hiebert & Wearne, 1996), including better preparation for future learning (Schwartz & Martin, 2004; Schwartz et al., 2011). However, there are two risks to inductive learning activities. First, it takes time to induce a pattern. Second, some individuals may never induce a useful symbolic structure (Gick & Holyoak, 1980). There is some evidence, however, that students who do not induce appropriate rules during discovery activities still show benefits from subsequent instruction (Kapur, 2008; Schwartz & Bransford, 1998).

### Knowledge from symbolic representations

Another approach is to begin with symbols, providing students with equations and asking them to practice them across several problems. There are several strengths to this approach. First, symbols are compact: an equation can express a whole range of values as a function. Previous research has shown that mathematics can help students learn more than verbal explanations (2005b; Schwartz et al., 2005a) and that beginning with symbolic representations can better promote transfer than starting with a range of concrete instances (Kaminski et al., 2009). To teach formulas, much of US instruction relies on clear lectures followed by practice problems (Third International Math and Science Study, Stigler & Hiebert, 2004). Researchers who champion direct instruction highlight these methods’ effectiveness in guiding students toward fluency (Chen & Klahr, 2003; Klahr & Nigam, 2004; Mayer, 2004). Furthermore, proponents of cognitive load theory posit that less-structured instructional methods unnecessarily overload students’ working memory (Kirschner et al., 2006; Sweller, 1988).

However, when students receive heavily symbolic instruction, they may not understand what the symbols represent. A risk of learning rules without an underlying conceptual model is that learners can have brittle knowledge of what is “right” and “wrong” without knowing why (Lehrer & Schauble, 2004; Lehrer et al., 2000). Students can memorize how to solve equations without learning why they work (Catrambone, 1998; Schwartz et al., 2011). Acquiring a purely symbolic understanding of a formula as a way to manipulate variables can also be problematic when approaching new situations; students with shallow understanding may be at risk of negative transfer, not recognizing when to apply an idea directly and when they need to modify it to fit a new situation. When students have well-rehearsed procedures, they can often miss key changes in problems, relying on perceptual similarity to help them determine what to do. For example, elementary-school children often make errors on equivalence problems, overgeneralizing their routines for addition (Knuth et al., 2006; McNeil & Alibali, 2004, 2005; Sherman & Bisanz, 2009). Students may also rely on other surface features, such as the cover story in word problems, and overgeneralize accordingly when their understanding of equations is not linked to the real-word contexts they represent (Ross, 1987).

### Instruction for transfer

It may be possible to integrate the two approaches through instruction for transfer. Because students need to connect a formula to the structures of the referent and map symbols into the context, such instruction aims to balance empirical investigation of new ideas and teaching symbolic forms. Many research-based instructional methods leverage this combination of direct instruction and discovery learning, in approaches such as schema-broadening instruction (e.g., Jitendra et al., 2015), problem-based learning (e.g., Loyens et al., 2011), and relational learning (Richland et al., 2012). Furthermore, there is evidence that beginning with an empirical search for structure can be a productive preliminary step before receiving direct instruction to promote transfer, in investigations of learning about ratios, statistics, and physics (Kapur, 2008, 2014; Schwartz & Martin, 2004; Schwartz et al, 2011).

In this paper, we investigate the role of searching for an equation before receiving direct instruction about an algebra problem. Our focus is on measuring students’ abilities to generalize appropriately to new contexts. Previous work on analogical transfer and transfer often focuses on situations where the context of a problem is changed and students need to recognize how new surface features map onto previous features, thus lifting and reapplying the same underlying deep structure to a new problem (e.g., Barnett & Ceci, 2002; Gentner & Markman, 1997; Gick & Holyoak, 1983; Reed, 1993). In contrast, we investigate a situation where the surface features are similar, but elements of the deep structure have changed, a different kind of challenge people face extending what they know to new situations (Schwartz et al., 2012). To answer our generalization questions appropriately, we think two steps are essential. First, people need to realize they should not overgeneralize. They need to understand the learned solution deeply enough to recognize what features of the context can vary while maintaining or violating conditions of applicability. The second step entails generating a new structure to accommodate the new question. In a mathematical task, this often involves finding a new equation that builds on a known solution.

Appropriate generalization is a challenging aspect of transfer (Ellis, 2007). People may not realize that the deep structure has changed and continue to use previously learned routines, showing negative transfer (Hutchins et al., 2020; Singley & Anderson, 1989). Even if they do realize that their previous routines are incorrect, they may not spontaneously produce new generalizations. People can be “overzealous” with their application of ideas across contexts when they do not recognize more efficient or appropriate alternatives (Schwartz et al., 2012). Forestalling this problem is important if the ultimate goal of education is to create adaptive expertise (Hatano & Inagaki, 1986; Schwartz et al., 2005a, 2005b). Thus, in Experiment 1, we contrasted negative transfer following instruction for transfer using search as compared to traditional tell-and-practice instruction. In Experiment 2, we augmented the instruction-for-transfer approach by giving learners a better idea of the goal of their inductive search, i.e., creating a formula. In Experiment 3, we used an enriched tell-and-practice condition.

### Sketching and diagrams

In Experiment 2, we also sought to understand the role of diagrams and drawing in avoiding negative transfer. Drawing sketches helps students visualize, elaborate on, and understand STEM concepts, which facilitates problem solving (Arcavi, 2003; Montague, 1998; Ruchti & Bennett, 2013; Van Garderen, 2006). The process of drawing aids in students’ understanding of the concepts underlying a new problem and reveals to teachers which concepts a student may misunderstand (Ruchti & Bennett, 2013). Self-directed drawing can be helpful for learning about scientific ideas (Ainsworth et al., 2011; Gobert & Clement, 1999; Leopold & Leutner, 2012; Van Meter, 2001). Sketching has been shown to outperform summarizing for learning about spatial domains such as biological mechanisms (Sheredos & Bechtel, 2017), geoscience (Gagnier et al., 2017; Garnier et al., 2017; Jaeger et al., 2018), and chemistry (Cooper et al., 2017).

While an extensive literature has detailed the role of diagrams in problem solving (e.g., Court, 1993; Larkin & Simon, 1987; Van Heuvelen, 1991), we investigate the role of prompting students to create their own sketches as a tool for algebraic problem solving. There is little prior research on sketching in this context. Based on findings about multiple representations in mathematics problem solving (Brenner et al., 1997; Hegarty & Kozhevnikov, 1999; Kieran, 1992), we thought that sketching in service of generating and solving an algebraic equation might help learners to create an additional representation.

## The polygon perimeter problem

**Fig. 1:**
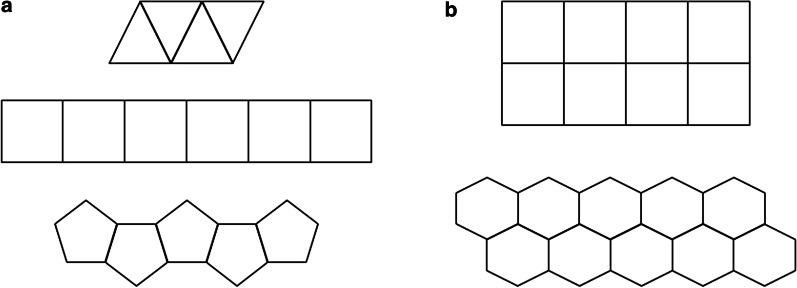
Study materials. **a** Sample figures used in the learning trials. Figures consisted of single rows of 3–10 shapes ranging from 3 sides (triangles) to 6 sides (hexagons). **b** Figures used in two transfer questions. In both arrangements, the perimeter formula for a single row of shapes must be adapted

Our task involved learning of an abstract rule for the perimeter of a row of shapes. The polygon perimeter problem is a growth pattern problem in which the solver’s task is to determine the perimeter of a row of regular polygons arranged in a single line (see Fig. 1a). The polygon perimeter problem has been used in mathematics education research as an example of growth pattern problem that allows a general abstract solution to be built from a range of possible contexts (e.g., Driscoll, 1999; Koellner et al., 2007). In our task, stimuli consisted of shapes ranging from three sides (triangles) to six sides (hexagons) in rows of 3–10 shapes. The total perimeter of the row varies linearly as a function of the size and number of shapes. Perimeter can be expressed in formulas that simplify to this format: Perimeter = (*s* − 2)*n* + 2, where *s* represents the number of sides per polygon and *n* represents the number of polygons in the row. For example, for the middle figure in Fig. 1a, *s* equals 4 because the individual polygons in the row are squares with four sides each and *n* equals 6 because there are six squares in the row. To compute the perimeter, a solver should first subtract 2 from *s* to account for the 2 sides of each shape that overlap on the interior edges and do not contribute to the outside perimeter edges of the figure. The resulting value (2) is multiplied by the number of shapes in the row (6), and then 2 is added to this product to represent the final two sides that make up the left and right edges of the row. The perimeter value is 14.

We tested the effects of applying a given formula or generating the formula from a diagram. Participants in the search condition received the formula afterward, so our research isolated the effects of beginning with a formula versus beginning with an inductive search and later receiving direct instruction. Of special interest was the effect on participants’ abilities to generalize appropriately to new problem types. We examined both steps of this process: avoiding overgeneralization and correct adaptation. Participants practiced with one row of shapes, and in our generalization transfer questions (see Fig. 1b), there were two rows of shapes. For these arrangements, the perimeter formula for a single row of shapes must be adapted. For example, for a figure with two rows of squares, 3 internal sides must be subtracted, modifying the first part of the formula to (*s* − 3). Subsequently, there are 2 additional edges to consider (one on each of the left and right sides), so the formula should be modified to (*s* − 3)*n* + 4. Substituting 4 and 8 for *s* and *n,* respectively, would result in 12, the perimeter of the two-row figure.

## Experiment 1

Experiment 1 investigated the optimal timing of direct instruction. We hypothesized that direct instruction and practice would lead to overgeneralization, and that inventing a formula would help avoid it. An open question was whether there is benefit for searching for a formula but not finding it, as found in productive failure research (Kapur, 2008).

### Method

#### Participants

Seventy-two participants were recruited through a large state university’s undergraduate psychology pool. Average age was 20.11 years old (SD = 2.45). All participants had taken algebra and geometry courses, but fewer had taken trigonometry, precalculus, or calculus courses. There were no significant differences in age, gender balance, or mathematics course experience between conditions. For more details, see Appendix (Table 11).

#### Design and procedure

There were 3 learning blocks (18 trials each) followed by transfer measures. The instructions of each learning block were manipulated such that participants spent time using different strategies to solve the problems. Participants were randomly assigned to condition: search or tell-and-practice. Each participant completed the study individually over approximately 45 min. All participants began by counting the perimeter in block 1. In block 2, participants either searched for a formula (search condition) or were told the formula (tell-and-practice condition). All participants were told the formula in block 3. Finally, participants completed generalization measures after the learning phase.

##### Learning Blocks

First, all participants completed a block of problems where they simply counted the number of sides to determine the perimeter. There were no condition differences in the instructions provided for block 1.

The second block differed by condition. Individuals in the search condition were asked to search for an algebraic rule for perimeter that can be used with all shapes and all numbers of shapes. They were asked to indicate when they had determined the rule to the experimenter verbally and the trial number on which they found the rule was recorded. After finding the rule, these participants were instructed to use it to solve for the perimeter on the remaining problems in the second block. Participants in the tell-and-practice condition were given the perimeter formula, (*s* − 2)*n + 2, at the beginning of the second block and asked to calculate the perimeter using this formula. The variables were defined in the instructions as the number of sides and number of shapes, but the relationship described by this function was not explained.

For block 3, all participants received the perimeter formula and were instructed to apply it to find the perimeter on these 18 trials. For participants in the tell-and-practice condition, the instructions for this block of trials were identical to the instructions in block 2. A complete list of the instructions is available on the Open Science Framework (OSF) repository for this project, which can be found at https://osf.io/3s8ay/?view_only=dea083debf6145df87591037f5ed917f.

#### Measures

##### Learning phase measures

We noted whether participants found a formula in the search conditions, and whether the formula was algebraic. For a formula to be considered algebraic, it must be able to be used without the referent present, i.e., it must be abstract. As an example, a formula that entailed counting the top and side edges of the figure and multiplying that total by 2 would not be considered algebraic because it still requires the solver to do a significant amount of counting using the figure. In contrast, a formula such as (*s* * *n*) − (*s* − 1) * 2 can be solved without a figure present, indicating that it is algebraic.

##### Generalization measures

After the three learning blocks, all participants answered four generalization measures. In the first two problems, the pattern was modified such that two rows of shapes made up the figure as shown in Fig. 1b. To correctly determine the perimeter, participants could count the number of outside edges or use a modified algebraic formula.

A second experimental factor was manipulated between-subjects at the time of the transfer questions to explore the nature of participants’ ability to adapt to new contexts; two forms of the transfer question were used. Form A stated, “Here is a new pattern. How would you solve this problem to find the perimeter?” In this question form, participants were asked to determine the perimeter with no guidance about strategy and no provided formula. In contrast, Form B included the formula and asked participants to *use or adapt* it to solve for the perimeter. The wording for Form B read, “Here is a new pattern and here is the formula you just used. [printed formula] Please *use or adapt* the formula you used before to solve this problem to get the perimeter.”

This difference in wording allows the experiment to explore an aspect of transfer. With Form A, participants can spontaneously generalize their own solution or our formula, or choose a different method, such as counting. Because Form B explicitly asks participants to use or adapt the provided formula, it tests whether participants can adapt a formula that may be different than their own methods. This is especially relevant in the Search condition, where some individuals could generate alternative forms of the perimeter solution during the learning phase of the study.

##### Coding scheme

Participants’ responses to the two transfer questions fell into three categories as described in Table 1. The “adapt” code applies to strategies that resulted in a formula with modified parameters that could be generalized to a different number of shapes arranged in the same double-row pattern. “Overgeneralizing” the formula was defined as directly applying the previously used formula to the new context without accounting for the new parameters posed by the different arrangements of shapes. This strategy resulted in an incorrect answer. “Other” strategies were not algebraic and did not result in a generalizable formula that could be used with a different number of shapes. Responses could only receive one code. If a participant adapted the formula and counted to double-check his or her solution, the response was coded as adapt. Interrater reliability for the eight squares and ten hexagons tasks was *κ* = 0.85 and *κ* = 0.92, respectively.

**Table 1:** Transfer question strategy codes and sample responses

Strategy code	Sample response
Adapt	“Number of sides minus 2 … I have to change that. The number of sides minus 4 times the number of shapes plus 6”
Overgeneralize	“I’d start by counting the number of shapes. It’s 2 rows of 5 so 10. The shapes are hexagons, so 4 times 10 is 40 plus 2 is 42”
Other	Counting, skip-counting, or other solutions

##### Visualization and formula comprehension measures

After the first two transfer questions, we included a two-part task that asked students to compute the perimeter for (a) a figure comprised of 100 squares in a single row and (b) a figure comprised of two rows of 100 squares each. We consider this a visualization measure, because participants received no diagrams and thus were required to visualize the figures. Participants’ responses were coded as overgeneralizing if they involved directly using the provided formula to calculate the perimeter for two rows of 100 squares (substituting 200 for the total number of shapes, ignoring the additional overlapping sides). Interrater reliability on this coding scheme was *κ* = 0.93. Table 3 shows participants’ approaches by condition.

Finally, we included a measure asking participants why we subtract 2 in the perimeter formula to gauge their understanding of how the formula related to the referent. Participants’ responses were coded for whether they included a reference to the idea of overlapping, shared, or internal sides that are not counted in the perimeter. This was used to measure comprehension of the formula. For the complete measures, see the OSF repository.

### Results

#### Learning task results

The majority of participants in the search condition (72%) found a formula. However, many of these formulas were not algebraic, but instead very closely tied to the referent. Formulas were coded as algebraic if they did not involve counting the entire perimeter and were applicable to all shapes and numbers of shapes. Only 17% of the search participants found an algebraic formula.

Response times on block 1 did not differ by condition (*M*_Search_ = 14.7 s, SD_Search_ = 10.8; *M*_Tell-and-practice_ = 14.1 s, SD_Tell-and-practice_ = 10.1; *t* = 0.95, *p* = 0.34). On block 2, tell-and-practice participants applying the formula had comparable average response times per trial, *M*_Tell-and-practice_ = 13.7 s, SD_Tell-and-practice_ = 9.6. In contrast, search participants were significantly slower with high variability, *M*_Search_ = 44.4 s, SD_Search_ = 55.8 (*t* = 13.59, *p* < 0.01). Tell-and-practice participants also had significantly lower average response times per trial on the third block as compared to search participants, likely an effect of practicing the formula (*M*_Tell-and-Practice_ = 9.8 s, SD_Tell-and-Practice_ = 5.4; *M*_Search_ = 12.7 s, SD_Search_ = 9.7; *t* = 6.71, *p* < 0.01).

#### Transfer task results

##### Two rows generalization measures

First, we report accuracy, and then we focus on strategy use. Table 2 shows the accuracy results by condition and question form. Chi-square tests found a significant relation between these factors and transfer accuracy for the eight squares measure (*χ*^2^ (3, *N* = 72) = 11.25, *p* = 0.01) and ten hexagons measure (*χ*^2^ (3, *N* = 72) = 8.95, *p* = 0.03). Adjusted standardized residuals were computed to determine the contribution of each condition and question form combination to the obtained chi-square values. A residual greater than ± 2 indicates a lack of fit with the null hypothesis that accuracy was at chance. Tell-and-practice condition participants given question Form B (provided the formula on the transfer tasks) had an adjusted residual of − 3.3 for the squares task and − 2.9 for the ten hexagons task, indicating that this combination of condition and question form performed worse than would be expected by chance. The difference by condition (collapsing across question form) was marginally significant for the eight squares question (*χ*^2^ (1, *N* = 72) = 3.60, *p* = 0.06) and the ten hexagons question (*χ*^2^ (1, *N* = 72) = 2.83, *p* = 0.09).

**Table 2:** Participants’ accuracy and strategy use as a function of condition and question form

	Eight squares				Ten hexagons			
	Accuracy (%)	Overgeneralize^a^ (%)	Other^a^ (%)	Adapt^a^	Accuracy (%)	Overgeneralize^a^ (%)	Other^a^ (%)	Adapt^a^
Tell-and-practice (*N* = 36)								
Form A	67	33	67	0	50	33	67	0
Form B	22	78	22	0	11	83	17	0
Search (*N* = 36)								
Form A	72	28	72	0%	55	39	56	6%
Form B	55	39	44	17%	44	44	44	11%

^a^Cells represent the percent of participants in each condition who used that strategy

Table 2 shows the strategy results by condition and question form. Chi-square tests found a significant relation between these factors and transfer strategy use for the eight squares measure (*χ*^2^ (3, *N* = 72) = 11.25, *p* < 0.005) and ten hexagons measure (*χ*^2^ (3, *N* = 72) = 14.65, *p* = 0.02). The tell-and-practice, question Form B condition had adjusted residuals of 3.3 for overgeneralizing on the eight squares task and ten hexagon task and − 2.9 for “other” strategies on both tasks. These residuals indicate that this combination of condition and question form were more likely to overgeneralize than would be expected by chance.

##### Visualization measure

The visualization measure included two parts. First, participants were given the formula and asked to compute the perimeter for a row of 100 squares. Next, participants were asked to compute the perimeter for a figure made up of two rows of 100 squares. The likelihood of overgeneralizing by condition is marginally significant (*χ*^2^ (1, *N* = 72) = 3.57, *p* = 0.06) (Table 3).

**Table 3:** Visualization measure strategies by condition

	Overgeneralize (%)	Correct (Add 2) (%)	Double (%)	Misc (%)
Tell-and-practice (*N* = 36)	64	25	8	3
Search (*N* = 36)	42	39	8	11

Cells represent the percent of participants in each condition that used that strategy

##### Formula comprehension measure

Participants’ responses were coded for whether they included a reference to the idea of overlapping, shared, or internal sides that are not counted in the perimeter. In general, participants performed quite well on this question. 83.3% of participants in the search condition got this question correct while 80.6% of participants in the tell-and-practice condition got this correct. This difference was not statistically significant between the conditions (*χ*^2^ (1, *N* = 72) = 0.94, *p* = 0.76). Anecdotally, it is possible that answering this question helped participants to understand the formula.

##### Transfer among search condition participants

We separated participants in the search condition into two groups: those who did and did not successfully invent a formula. The rate of inventing was low, which affords us the ability to do a more direct comparison of the effects of inventing a formula (see Table 4). On all three tasks, tell-and-practice participants were most likely to overgeneralize. Participants who did invent an algebraic formula were the least likely, and participants who searched for a formula but did not arrive at an algebraic formula were in the middle. We compared the rate of overgeneralizing among tell-and-practice participants to search participants who did not find a formula to investigate the possibility of productive failure. The trend is consistent across all three tasks, but we did not find statistically significant evidence of an advantage of trying to search for the eight squares task (*χ*^2^ (1, *N* = 66) = 3.26, *p* = 0.07), the ten hexagons task (*χ*^2^ (1, *N* = 66) = 0.894, *p* = 0.34), or the 200 squares task (*χ*^2^ (1, *N* = 66) = 1.97, *p* = 0.16).

**Table 4:** Likelihood of overgeneralizing by condition and formula-finding

	Eight squares (%)	Ten hexagons (%)	200 squares (%)
Tell-and-practice (*n* = 36)	56	58	64
Search			
Found algebraic formula (*n* = 6)	17	17	17
Did not find formula (*n* = 30)	33	47	47

Cells represent the percent of participants in that condition who overgeneralized

### Discussion

We observed few algebraic equations among participants in the search condition. Instead, many students created procedures that relied on constants and counting rather than variables. Thus, when given a new problem, these students were likely to continue counting and using other non-general solution methods. We did observe condition differences, particularly on question Form B, which asked students to use or adapt what they had learned (a typical feature of assessments). Providing the formula on the transfer test was especially associated with overgeneralizing for students in the tell-and-practice condition. Being told a formula and then being asked to apply it in another context runs the risk of overgeneralizing, as one may not question whether the previously learned routine can be directly applied. Without a formula, participants were more likely to reason about the problem directly, using counting or other strategies.

## Experiment 2

In Experiment 2, we sought to remedy an issue in Experiment 1 and to extend our line of inquiry with a new instructional condition. In Experiment 1, we observed that participants had difficulty with finding an *algebraic* formula. As reported, many participants created non-algebraic lists of procedures or other non-generalizable methods for finding the perimeter that relied on counting. In Experiment 2, we added more support to our definition of a formula by providing examples in the beginning of the second block for participants in the Search conditions.

We also investigated the role of diagrams and sketching. Some participants received diagrams as in the previous study, and others were asked to sketch the shape figures. We crossed this sketching manipulation with the search versus tell-and-practice manipulation. With the search instructions, our hypothesis was that sketching may help people see which parts of the shapes contribute to the perimeter, and then be more likely to find a formula. With tell-and-practice instructions, we thought sketching may help people connect the provided formula to the figure. We predicted that the sketching conditions would be less likely to overgeneralize than participants given diagrams for each problem.

### Method

#### Participants

Undergraduate participants (*n* = 161) were recruited through the same undergraduate psychology participant pool and randomly assigned to one of four between-subjects conditions: tell–diagram (*n* = 38), tell–sketch (*n* = 39), search–diagram (*n* = 39), and search–sketch (*n* = 37). Three individuals were dropped from analyses for miscounting perimeter on over half of the block 1 trials. Additionally, we dropped five individuals who arrived late to their study sessions and did not complete the post-tests. One-hundred-fifty-three participants remained in the analyses. See Appendix (Table 12) for a summary of the demographics.

#### Materials and procedure

The procedure for Experiment 2 was very similar to Experiment 1. However, participants solved the problems on pencil and paper instead of using a computer, completing three separate problem packets, the transfer problems, and a demographics sheet. No additional tools, such as calculators, were provided. The entire session took approximately 1 h. We allowed up to four participants per timeslot. Individuals worked at opposite corners of a large conference room and did not collaborate; we analyze these data at the individual level. A researcher remained in the room for each session, distributing and collecting materials from each individual. Researchers instructed the participants to complete each page in order, without referring to any previous pages. Experimenters answered clarifying questions but did not provide hints or assistance to participants. For example, researchers answered questions such as “what does ‘perimeter’ mean?” and “do I need to sketch for this part?” but did not answer “what is the formula?” or make any comments about individuals’ progress.

Trials were again presented in a fixed randomized order. Because of the additional manipulation, the number of trials per study block was decreased from 18 to 13, which still allowed for each item to be shown at least once. Seven items were shown twice and these repeat items were distributed across shapes and numbers of shapes. Following the study blocks, we assessed participants’ transfer and understanding of the formula using the same measures as in Experiment 1 (see OSF for complete instructions and measures). Finally, participants completed a paper-based demographics questionnaire.

#### Design

The study used a 2 × 2 between-subjects design, crossing the previous manipulation (search vs. tell-and-practice) with a sketching manipulation (sketch vs. diagram). Participants either referenced shapes printed on a page (diagram conditions) or read descriptions of shapes to sketch (sketch conditions).

##### Sketching manipulation

The sketching manipulation was introduced during block 1 of the study. Participants in sketch conditions received descriptions of figures to draw (e.g., “three triangles in a row”). These individuals also received a reference sheet showing how to draw each type of shape in a row (see OSF repository). In block 1, sketch participants were instructed to sketch the figures described and count the outside edges. In the subsequent blocks, no explicit instructions about sketching were given, but participants continued to receive descriptions of the figures instead of diagrams. Participants in the diagram conditions received images for all three blocks as in Experiment 1.

##### Search conditions: instruction about formulas

To clarify the task, participants in the search groups received a sheet explaining algebraic formulas before beginning Block 2. The sheet explained that a formula is a set of mathematical steps taken to find the perimeter without counting each edge, which works for all types of shapes (e.g., a formula that only works for triangles would not count). Participants were free to ask questions before moving on, and researchers limited answers to those about formulas in general; researchers did not reveal or hint at steps to the target formula.

#### Measures

Experiment 2 used the same measures and similar coding schemes as Experiment 1. Participants first completed two transfer questions asking for the perimeter of two-row figures (squares and hexagons). All participants received the formula and were asked to *use or adapt the formula to find the perimeter* (Form B). Responses to these questions were coded for accuracy and strategy. Because of the nature of small-group data collection (as compared to individual think-aloud protocols), we could not always distinguish between correct adaptation of the formula and counting. Both strategies indicated that a participant did not use the given formula for these questions. Interrater reliability was *κ* = 0.96 on the eight squares task and *κ* = 0.96 on the ten hexagons task.

Next, participants completed the two-part question asking participants to determine the perimeter of a row of 100 squares and two rows of 100 squares. As in Experiment 1, responses were coded for accuracy and strategy use. Interrater reliability on the coding scheme for this question was *κ* = 0.75.

Finally, participants’ knowledge of the formula was probed by again asking, “What does the ‘− 2’ in the formula represent? Why do we subtract two?” Responses were coded for whether they attributed the “− 2” to the overlapping sides in the row.

### Results

#### Learning task results

We coded individuals as having found a formula if they circled “yes” for the question “Did you use a formula to find the perimeter?” and recorded the formula they noted during the search packet. Sixty-four participants (84% of the search conditions) reported that they had found a formula. There was no association between finding a formula and accuracy on the eight squares (*χ*^2^ (1, *N* = 76) = 2.83, *p* = 0.09), ten hexagons (*χ*^2^ (1, *N* = 76) = 0.01, *p* = 0.92), or 200 squares (*χ*^2^ (1, *N* = 76) = 1.68, *p* = 0.43) questions. Separately, we coded whether each formula was algebraic. Of the 64 participants who stated that they found a formula, 36 found an algebraic formula, i.e., 48% of the participants in the search conditions found an algebraic formula.

We coded whether the formulas that participants found were algebraic and we compared accuracy among participants who did and did not find algebraic formula during the learning phase and accuracy on the transfer measures. There was a positive relationship between finding an algebraic formula during the learning phase and accuracy on the eight squares transfer question, *χ*^2^ (1, *N* = 76) = 4.6, *p* = 0.03. There was no relationship between finding an algebraic formula and accuracy on the ten hexagons (*χ*^2^ (1, *N* = 76) = 0.553, *p* = 0.46) or 200 squares (*χ*^2^ (1, *N* = 76) = 4.66, *p* = 0.10) questions.

The amount of time participants spent on the learning blocks varied by condition, with searching and sketching both requiring more time. On average, participants in the tell–diagram condition took 16.34 min (SD = 5.98) and tell–sketch participants took 25.78 min (SD = 9.63). The search conditions took longer; search–diagram participants took an average of 29.51 min (SD = 9.72) to complete the learning blocks with the average for search–sketch at 34.35 min (SD = 8.83).

##### Effects of sketching on finding a formula

We also investigated whether sketching or seeing diagrams affected participants’ ability to find a formula during the search task. Seventy-eight percent of participants in the search–sketch condition and 90% of search–diagram participants reported finding a formula. There was no significant effect of condition on participants’ likelihood of finding a formula (*χ*^2^ (1, *N* = 76) = 1.84, *p* = 0.17) or on their likelihood of finding an algebraic formula, *χ*^2^ (1, *N* = 76) = 2.63, *p* = 0.11. The process of visualizing and sketching a row of shapes did not facilitate formula induction.

#### Transfer task results

##### Two rows generalization measures

Table 5 shows the accuracy results by condition. Chi-square tests found a significant relation between these factors and transfer accuracy for the eight squares measure (*χ*^2^ (3, *N* = 153) = 18.04, *p* < 0.001) and ten hexagons measure (*χ*^2^ (3, *N* = 153) = 10.87, *p* = 0.01). On the squares question, adjusted standardized residuals of 3.8 for the search–diagram condition and − 3.1 for the tell–diagram conditions indicate that searching for a formula and receiving a diagram was particularly associated with correct performance and that participants who saw a diagram and were told the formula were particularly likely to be incorrect. Similar adjusted standardized residuals for the ten hexagons question (2.1 for search–diagram and − 3.1 for tell–diagram) indicate that this pattern is stable across both question types. Separate hierarchical log-linear models for the eight squares and ten hexagon tasks indicate that a third-order interaction among search/tell-and-practice condition assignment, sketch/diagram assignment, and accuracy is significant for both the eight squares task (*χ*^2^ (1) = 9.55, *p* = 0.002) and the ten hexagons task (*χ*^2^ (1) = 5.77, *p* = 0.02).

**Table 5:** Post-test task accuracy by condition

	Eight squares (%)	Ten hexagons (%)	200 squares (%)	Why − 2? (%)
Tell-and-practice				
Diagram (*n* = 37)	29	18	19	63
Sketch (*n* = 39)	49	44	31	66
Search (*n* = 36)				
Diagram (*n* = 39)	77	54	25	75
Sketch (*n* = 38)	49	43	20	81

Examining strategy use supports the accuracy results. As shown in Table 6, there was a significant effect of condition assignment on participants’ likelihood of overgeneralizing for the eight squares task (*χ*^2^ (3, *N* = 153) = 18.02, *p* < 0.001) and the ten hexagons task (*χ*^2^ (3, *N* = 153) = 21.25, *p* < 0.001). Adjusted standardized residuals of − 3.7 for the search–diagram condition and 3.2 for the tell–diagram condition indicate that these two conditions overgeneralized less often and more often than expected by chance on the eight squares task, respectively. The same pattern was observed for the ten hexagons task (adjusted standardized residual = − 3.6 for search–diagram and 3.9 for tell–diagram). Separate hierarchical log-linear models for the squares and hexagon tasks indicate that a third-order interaction among search/tell-and-practice condition assignment, sketch/diagram assignment, and likelihood of overgeneralizing is significant for both the squares task (*χ*^2^(1) = 9.04, *p* = 0.003) and the hexagons task (*χ*^2^(1) = 10.74, *p* = 0.001).

**Table 6:** Participants’ likelihood of overgeneralizing as a function of condition

	Eight squares (%)	Ten hexagons (%)	200 squares (%)
Tell-and-practice			
Diagram (*n* = 37)	68	74	68
Sketch (*n* = 39)	49	46	39
Search			
Diagram (*n* = 39)	21	21	19
Sketch (*n* = 38)	47	44	31

##### Visualization measure

Table 6 indicates the percent of participants in each condition who overgeneralized on this question, directly using the provided formula for both one row and two rows of squares. A chi-squared analysis denotes a significant effect of condition, *χ*^2^ (3, *N* = 147) = 19.24, *p* < 0.001. Examining the adjusted standardized residuals indicates that participants in the search–diagram condition were again less likely to overgeneralize than expected (adjusted standardized residual = − 2.8) and individuals in the tell–diagram condition were more likely to overgeneralize than expected by chance (adjusted standardized residual = 4.0). A third-order interaction among search/tell-and-practice condition assignment, sketch/diagram assignment, and overgeneralizing on this measure was found to be significant via hierarchical log-linear analysis (*χ*^2^(1) = 6.47, *p* = 0.01).

##### Formula comprehension measure

Similar to Experiment 1, participants were quite accurate at answering this question; across condition, 72% of participants were correct. There was no effect of condition on accuracy, *χ*^2^ (3, *N* = 153) = 3.75, *p* = 0.29. There was a significant positive relationship between this question and accuracy on the eight squares (*χ*^2^(1) = 4.17, *p* = 0.04) and ten hexagons (*χ*^2^(1) = 7.82, *p* = 0.005) questions.

##### Transfer among search condition participants

Table 7 shows the likelihood of overgeneralizing on the three generalization tasks by condition and formula-finding success. Again, participants who found a formula were the least likely to overgeneralize. Participants in the Search condition who did not successfully find an algebraic formula were more likely to overgeneralize and participants in the tell-and-practice condition were most likely to overgeneralize. We again compared the likelihood of overgeneralizing in the tell-and-practice condition to participants who searched but failed to find a formula. We did not find statistical evidence to support the idea of productive failure on the eight squares task (*χ*^2^ (1, *N* = 114) = 1.58, *p* = 0.21), but we found marginally significant differences on the ten hexagons task (*χ*^2^ (1, *N* = 114) = 3.64, *p* = 0.06) and 200 squares task (*χ*^2^ (1, *N* = 114) = 3.46, *p* = 0.06).

**Table 7:** Likelihood of overgeneralizing by condition and formula-finding

	Eight squares (%)	Ten hexagons (%)	200 squares (%)
Tell-and-practice (*n* = 76)	58	60	53
Search			
Found algebraic formula (*n* = 36)	19	23	15
Did not find formula (*n* = 41)	46	41	34

Cells represent the percent of participants in that condition who overgeneralized

### Discussion

Comparing the two diagram conditions, we observe that participants who search for a formula are consistently less likely to overgeneralize it across measures.

Search participants were more likely to find an algebraic formula than in Experiment 1, which indicates that providing additional information about formulas seemed to help participants understand how to create formulas. It is also possible that completing the task on paper rather than via computer-facilitated searching; this question could be studied in future work.

Across all three generalization measures, a consistent pattern emerged: sketching seemed to hinder performance when coupled with the search instructions and help performance among the tell-and-practice condition. Because we primarily aimed to explore potential relationships between searching and overgeneralization, we did not continue the sketching manipulation into the next experiment, as it only improved performance in the tell-and-practice condition.

## Experiment 3

In Experiment 3, we sought to control time on task, which was previously uncontrolled. In previous experiments, participants in the search conditions spent more time on block 2 than tell-and-practice participants, because searching for a formula was more time-consuming than applying a given formula. It is possible that simply spending more time on the problems led to the lower rates of negative transfer, instead of the instructional manipulation. Thus, we required all participants to spend the same amount of time during the learning phases in Experiment 3.

We also added more instructional support to the tell-and-practice condition by explaining the function of each operation in the formula and visually tying each step to the geometric figure. Tell-and-practice condition participants received this direct instruction in blocks 2 and 3; search condition participants received it in block 3. More elaborate direct instruction may be more similar to classroom experiences than the direct instruction provided in Experiments 1 and 2. We preregistered our study design and analysis plan for Experiment 3 (see https://osf.io/3s8ay/?view_only=dea083debf6145df87591037f5ed917f).

### Method

#### Participants

Using the pwr package in R, we conducted a power analysis to determine a sufficient sample size for this experiment. To achieve a power of 0.9 with 1 *df* and a significance level of 0.05, we found that we needed 64 total participants to detect an effect size as strong as the effect observed between the two diagram conditions in Experiment 2. We recruited several extra participants to ensure that we would have sufficient power even if any participants were dropped and also to have more sensitivity.

Seventy-four participants were recruited through the same undergraduate psychology participant pool and randomly assigned to one of two between-subject conditions. Of them, four were excluded from analyses: one was under 18 years old, one did not complete the transfer tasks, and two struggled substantially with the first block of counting problems. One additional participant did not complete the demographics survey and was excluded from descriptive statistics. Sixty-nine participants remained between the tell-and-practice and search groups (57 women, 12 men). See Appendix (Table 13) for a summary of the demographics.

#### Materials

Participants completed written packets for each study block. The tell-and-practice packet (received in blocks 2 and 3 in the tell-and-practice condition, and in block 3 in the search condition) was more elaborate compared to previous experiments. This packet details step-by-step instructions about how the formula works, connecting each algebraic step to the figure. For instance, “We subtract 2 because 2 sides on each shape are not part of the perimeter.” See the OSF repository to compare the complete instructions for each condition in block 2. As in previous experiments, an example figure accompanied the tell-and-practice condition instructions.

#### Procedure

The entire session took approximately 1 h. We allowed up to four participants per timeslot, all being assigned the same condition. Experiment 3 followed the same 3-packet learning phase paradigm followed by a post-test packet of generalization questions. In this experiment, time on task was controlled; each participant spent 5 min on block 1, and 8 min each on blocks 2 and 3. Because of the time on task manipulation, the number of items per problem packet was increased to ensure continued practice. If participants finished early, the experimenter instructed them to check their work by redoing the problems.

Participants were randomly assigned to condition (search or tell-and-practice). All participants began by counting the perimeter in block 1. In block 2, participants either searched for a formula (search condition) or were told the formula (tell-and-practice condition). All participants were told the formula in block 3. After the learning phase, participants completed transfer measures and a demographics sheet.

#### Measures

As in the previous experiments, we recorded whether participants found a formula in the search conditions, and whether the formula was algebraic. We used the same measures as in Experiments 1 and 2. Participants completed two transfer questions asking for the perimeter of two-row figures (squares and hexagons). All participants received the formula and were asked to *use or adapt the formula to find the perimeter.* Responses to these questions were coded for accuracy and strategy. Interrater reliability was *κ* = 0.96 on the squares task and *κ* = 0.96 on the hexagons task.

Participants completed the two-part question asking participants to determine the perimeter of a row of 100 squares and two rows of 100 squares. As in Experiments 1 and 2, responses were coded for accuracy and strategy use. Interrater reliability on the coding scheme for this question was *κ* = 0.75. Finally, participants were asked, “What does the ‘− 2’ in the formula represent? Why do we subtract two?”. Responses were coded for whether they attributed the “− 2” to the overlapping sides in the row.

### Results

#### Learning task results

The majority of search condition participants (68%) found a formula during the search block. We analyzed the relationship between finding a formula during the learning phase and accuracy on the transfer measures. There was a relationship between inventing a formula and accuracy for the eight squares (*χ*^2^ (1, *N* = 69) = 7.27, *p* = 0.01), ten hexagons (*χ*^2^ (1, *N* = 69) = 5.72, *p* = 0.02), and the 200 squares (*χ*^2^ (1, *N* = 69) = 8.87, *p* < 0.01) measures.

We separately coded whether participants found an algebraic formula. Of the 23 participants who found a formula in the search condition, 19 of them (86%) found an algebraic formula. There was no relationship between finding an algebraic formula and accuracy on any of the post-test transfer measures; for the eight squares (*χ*^2^ (1, *N* = 69) = 0.20, *p* = 0.66), ten hexagons (*χ*^2^ (1, *N* = 69) = 0.03, *p* = 0.86), and the 200 squares (*χ*^2^ (1, *N* = 69) = 0.009, *p* = 0.92) post-test transfer measures.

#### Transfer task results

##### Two rows generalization measures

Table 8 shows the accuracy results by condition. Performance was quite similar between the two conditions. There was no significant effect of condition on accuracy for the eight squares or ten hexagons transfer questions; (*χ*^2^ (1, *N* = 69) and (*χ*^2^ (1, *N* = 69) = 0.61, *p* = 0.44), respectively.

**Table 8:** Post-test task accuracy by condition

	Eight squares (%)	Ten hexagons (%)	200 squares (%)	Why − 2? (%)
Tell-and-practice (*n* = 36)	72	56	39	69
Search (*n* = 34)	68	65	35	85

Participants’ strategies were coded as in the previously reported studies. See Table 9 for the likelihood of overgeneralizing on each problem. There was not a significant difference in likelihood of overgeneralizing by condition for the eight squares problem (*χ*^2^ (1, *N* = 69) = 0.01, *p* = 0.92) or the ten hexagons problem (*χ*^2^ (1, *N* = 69) = 1.27, *p* = 0.26). On the ten hexagons problem, 47% of search condition participants and 17% of tell-and-practice condition participants adapted the formula; search participants were significantly more likely to adapt the formula for this measure (*χ*^2^ (1, *N* = 69) = 6.15, *p* = 0.01). However, this effect was not statistically significant for the eight squares problem, with 53% and 33% of the participants adapting the formula in the search and tell-and-practice condition participants, respectively (*χ*^2^ (1, *N* = 69) = 2.0, *p* = 0.16). This may suggest that the effect of condition may be more pronounced on more challenging transfer questions.

**Table 9:** Participants’ likelihood of overgeneralizing as a function of condition

	Eight squares (%)	Ten hexagons (%)	200 squares (%)
Tell-and-practice (*N* = 36)	28	39	25
Search (*N* = 34)	29	23	26

##### Visualization measure

There was no significant effect of condition on overgeneralization on the 200 squares measure (*χ*^2^ (1, *N* = 69) = 0.02, *p* = 0.89). In both conditions, the incidence of overgeneralizing was quite low, as shown in Table 9.

##### Formula comprehension measure

Participants were generally accurate in answering the comprehension question; 78% of participants correctly indicated that the “− 2” in the formula corresponds to the shared sides. While search participants were more likely to be accurate, there was not a statistically significant effect of condition (*χ*^2^ (1, *N* = 69) = 2.49, *p* = 0.11). Accuracy on this comprehension question was marginally associated with higher performance on the squares question, (*χ*^2^ (1, *N* = 69) = 3.95, *p* = 0.05), but not significantly associated with performance on the hexagons question (*χ*^2^ (1, *N* = 69) = 2.28, *p* = 0.13).

##### Transfer among search condition participants

Table 10 shows the likelihood of overgeneralizing on the transfer tasks by condition and formula-finding success. When comparing participants in the tell-and-practice condition, participants in the search condition who found a formula, and participants in the search condition who did not find a formula, we found a significant effect of group membership on the 100 squares problem (*χ*^2^ (1, *N* = 69) = 6.73, *p* = 0.04) and the ten hexagons problem (*χ*^2^ (1, *N* = 69) = 9.17, *p* = 0.01). There was a marginal effect on the eight squares problem (*χ*^2^ (1, *N* = 69) = 5.06, *p* = 0.08). Participants who found a formula were the least likely to overgeneralize. We did not find evidence to support a productive failure argument in these findings; participants who searched but did not find a formula were not better prepared to transfer than participants who received direct instruction (and, in fact, were more likely to overgeneralize, though there may be selection bias in incoming math ability within this group).

**Table 10:** Likelihood of overgeneralizing by condition and formula-finding

	Eight squares (%)	Ten hexagons (%)	200 squares (%)
Tell-and-practice (*n* = 36)	28	39	25
Search			
Found algebraic formula (*n* = 23)	17	9	13
Did not find formula (*n* = 11)	55	55	55

Cells represent the percent of participants in that condition who overgeneralized

### Discussion

Study 3 was conducted to investigate the effectiveness of more elaborate tell-and-practice instruction in comparison with our search condition. We preregistered our study plan and we conducted analyses in the same manner as in the previous studies. These findings suggest that direct instruction can be effective for teaching formulas, especially when the instruction explains how each aspect of the formula works and when the formula is tied to a geometric referent.

Participants who searched and successfully found a formula were the least likely to overgeneralize on all 3 transfer problems, however. Tell-and-practice was not as strong a buffer against overgeneralizing as searching was, perhaps indicating that the participants who searched and found a formula had a deeper understanding of the mechanics of the formula.

## General discussion

In three experiments, we compared a typical instructional method, tell-and-practice, to inventing with examples before receiving direct instruction. In our first two studies, we consistently found that beginning with a search for a formula helped learners avoid negatively transferring the formula that they learned, although in Experiment 2 that effect did not hold when they also had to sketch. We suggest that search helps students connect the formula to the referent. However, in Experiment 3, we found that more elaborate direct instruction works almost as well as search, perhaps by tying mathematical procedures to the underlying concepts.

### Does searching affect generalization?

In the introduction, we outlined two steps involved in properly generalizing a formula to a new problem with a different underlying structure. First, we hypothesized that people need to notice that a previously helpful routine was no longer applicable and needed to modification. In our studies, we found evidence that this step was more likely to occur among people who first searched for a formula before receiving direct instruction about the steps of the formula. These participants were less likely to overgeneralize and continue using the formula when it was no longer appropriate. A second component of generalizing is that people need to determine how to adapt the formula to create a new formula.

Participants’ experience with mathematics was likely a factor in their success on the searching task. Some participants in Experiment 1 created non-algebraic formulas very tied to the referents and that lacked abstraction. These participants were more likely to overgeneralize than their counterparts who created algebraic formulas. Providing clearer task instructions helped to alleviate this issue in Experiments 2 and 3.

Even with scaffolding, some participants were more successful than others at inventing an algebraic formula. There may be stable individual differences in how likely people are to generate rules rather than focus on exemplars. McDaniel and colleagues (2014) found that people who generated rules to describe patterns in visual datasets were more likely to generalize to a novel categorization task and more likely to resist using idiosyncratic features in generalizing the trained category. Similarly, our participants who invented formulas were less susceptible to overgeneralizing based on surface features. Future work could investigate whether participants who invent algebraic formulas in geometry do the same in other spatial and geometric tasks.

Generally, our findings suggest that searching prior to direct instruction can help learners develop an understanding of the formula and mathematical processes such that they recognize a need for generalization and do not negatively transfer based on surface cues when it is no longer applicable (Schwartz & Martin, 2004). These findings align with research on concreteness fading, which suggest that beginning with more concrete instances and working toward an abstract representation can be an effective way of developing transferable knowledge (Fyfe et al., 2014; Goldstone & Son, 2005). Though there are some tradeoffs with efficiency during the learning phase, the benefits of this approach appear on future learning tasks. The scope of instruction, however, was not sufficient for a majority of learners to reach the second phase of generalization, correct adaptation. Future research could build on this work by providing instruction lasting more than a single session.

### Does sketching help?

A promising finding from Experiment 2 is that sketching helped people learn from direct instruction. However, in conjunction with the search instructions, it was more effective to provide learners with a diagram. Perhaps the combination of inventing and drawing a sketch was too taxing in terms of extrinsic cognitive load (Paas et al., 2003) for these participants. Alternatively, it may be that in tell-and-practice instruction, participants could think about how the given formula related to the individual sides of the shapes they were constructing during the sketching activity (Arcavi, 2003). In contrast, diagrams might help students search for a formula. Drawing a figure could be a detriment as it adds an additional step between each searching trial and could result in a switch cost whereby the processing of the formula and mathematics may be broken up (Wylie & Allport, 2000). This pattern points to the need for more research regarding sketching and problem solving under different instructional conditions.

### Limitations

One limitation of our investigation is that we did not include a pretest. Because we are interested in the nature of students’ inventing mathematical ideas for new problems for which they have not received previous instruction, we did not want to use a pretest that may have biased participants by providing them with an initial experience solving algebra problems involving geometric figures. We used mathematics class experiences as a proxy when possible, and we did not see an effect when comparing individuals who did or did not have experience taking calculus courses. Still, future research could include a pretest or individual differences measures as covariates to ensure that our results could not be explained by selection bias in incoming mathematics ability.

Additionally, we do not know how participants would have performed on the generalization tasks had they received feedback, as they would in a classroom. The stimuli themselves provided some intrinsic feedback—by counting, participants could realize that applying the formula was not correct. However, we do not know whether more direct feedback or subsequent direct instruction would have affected the pattern of results.

Because the learning task was self-paced, time on task was not equated across participants in Experiments 1 and 2. (In Experiment 3, we controlled time on task.) In Experiments 1 and 2, we equated the number of problems that all participants solved instead. Learners who were in the search conditions generally took longer to search for a formula than it took for participants to apply a given formula. However, the argument that total time on task is solely determinant of transfer success is undermined by the fact that, in Experiment 2, the condition that took the longest time was the search–sketch condition. If simply spending more time was beneficial, we would expect this condition to have the lowest rates of overgeneralizing.

Finally, a set of limitations relate to the generalizability of these findings to classroom contexts. We recognize that laboratory research with a relatively short intervention does not directly map to educational settings. We suggest that translational research that builds on these studies in authentic classroom contexts could be a productive step in the application of these findings. Considerations such as optimal ways of providing feedback during the search and generalization phases, ways to handle the idiosyncrasies of invented solutions, and creating tasks with multiple entry points for learners of different mathematical experiences would all come into play for translating this approach to schools.

### Implications

Despite the limitations, we see several implications of this work. Our most successful instructional condition included a blend of inventing and tell-and-practice, suggesting that a compromise between these two types of instruction may be especially effective at helping students gain deep understanding. With respect to avoiding incorrect overgeneralization, in all studies, those in the search condition who were able to generate an algebraic formula performed best. We found marginal and consistent evidence in support of productive failure over basic tell-and-practice instruction in Experiments 1 and 2, suggesting that simply searching for a formalism even without succeeding at creating an algebraic formula may be a worthwhile preliminary step before receiving formula-only direct instruction. However, in Experiment 3, those who received enhanced direct instruction performed on average better than those in the search condition who were unable to generate an algebraic formula.

We found evidence that searching was productive for resisting overgeneralizing, but that students may not be likely to find a modified formula with only one instance of a new pattern. Anecdotally, our brief formula comprehension question seemed to help participants connect the formula to the referent. Perhaps asking students to explain the steps of an equation in context could be fruitful in laying the groundwork for creating new abstractions.

Our results underscore the importance of focusing on transfer and generalization in assessments. Had we only focused on efficiency on a single problem type, we might have concluded that inventing and discovery learning are not worth pursuing because they are slower. However, by considering the overall efficiency across the learning task and the generalization task, it appears that the benefits of these more open-ended instructional techniques appear on future problem solving, to a strong degree over formula-only direct instruction, and to a lesser degree over direct instruction closely tied to the referent. Further, this generalization task extends the field’s investigation into transfer and the ways that learning tasks can prepare people for future learning.

## Conclusion

We offer a contribution to the goal of creating learners with adaptive expertise that can be generalized across contexts and applied in new learning situations. We extend previous research on inventing paradigms by incorporating a generalization task and identifying avoidance of overgeneralization as a first step toward adaptation. Across three studies, our findings suggest that instructional approaches that orient students to the spatial referents underlying symbolic formulas, such as encouraging students to search for deep structure prior to receiving direct instruction, can support students in stopping their routine processing and recognizing the need for adaptation. Ultimately, this understanding can help learners to not only know procedures but also know the boundary conditions that define the usefulness of their ideas.

## Data Availability

The preregistered study plan and datasets used during this study are available through the Open Science Framework online repository at the following link: https://osf.io/3s8ay/?view_only=dea083debf6145df87591037f5ed917f.

## Demographics by condition

See Tables 11, 12, and 13.

**Table 11:** Experiment 1 descriptive statistics by condition

	Age (SD)	Gender (% female) (%)	College year (SD)	Algebra (%)	Geometry (%)	Calculus (%)	Science or engineering major (%)
Tell-and-practice (*N* = 36)	20.00 (1.85)	64	2.36 (1.07)	100	100	53	14
Search (*N* = 36)	20.22 (2.96)	56	2.19 (1.01)	100	100	61	11

College year: 1 = freshman, 2 = sophomore, 3 = junior, 4 = senior

Algebra, geometry, calculus: % of participants who had taken this course

Science or engineering major: % of participants who were science or engineering majors

**Table 12:** Experiment 2 descriptive statistics by condition

	Age (SD)	Gender (% female) (%)	College year (SD)	Algebra (%)	Geometry (%)	Calculus (%)	Science or engineering major (%)
Tell-and-practice							
Diagram (*N* = 37)	20.32 (3.31)	79	2.30 (1.14)	100	100	45	16
Sketch (*N* = 39)	19.73 (1.34)	77	2.05 (0.89)	100	100	41	13
Search							
Diagram (*N* = 39)	19.15 (1.08)	73	1.97 (0.95)	100	100	59	22
Sketch (*N* = 38)	19.76 (2.07)	70	2.32 (1.04)	100	100	51	16

College year: 1 = freshman, 2 = sophomore, 3 = junior, 4 = senior

Algebra, geometry, calculus: % of participants who had taken this course

Science or engineering major: % of participants who were science or engineering majors

**Table 13:** Experiment 3 descriptive statistics by condition

	Age (SD)	Gender (% female) (%)	College Year (SD)	Algebra (%)	Geometry (%)	Calculus (%)	Science or engineering major (%)
Tell-and-practice (*N* = 36)	23.67 (10.44)	78	2.78 (1.01)	97	97	36	14
Search (*N* = 33)	21.39 (4.34)	88	2.87 (1.13)	100	100	58	15

College year: 1 = freshman, 2 = sophomore, 3 = junior, 4 = senior

Algebra, geometry, calculus: % of participants who had taken this course

Science or engineering major: % of participants who were science or engineering majors
